# Computational Development of Multi-Epitope Reovirus Vaccine with Potent Predicted Binding to TLR2 and TLR4

**DOI:** 10.3390/ph18111632

**Published:** 2025-10-29

**Authors:** Abdullah Al Noman, Abdulrahman Mohammed Alhudhaibi, Pranab Dev Sharma, Sadia Zafur Jannati, Tahamina Akhter, Samira Siddika, Kaniz Fatama Khan, Tarek H. Taha, Sulaiman A. Alsalamah, Emad M. Abdallah

**Affiliations:** 1 Department of Chemistry and Biochemistry, Kennesaw State University, Kennesaw, GA 30144, USA; abdullah.al.noman@g.bracu.ac.bd (A.A.N.); kanizfatamakhan2611@gmail.com (K.F.K.); 2 School of Pharmacy, BRAC University, Dhaka 1212, Bangladesh; sadiazafurjannati123@gmail.com (S.Z.J.); tahamina.akhter@g.bracu.ac.bd (T.A.); samirasiddikaeva@gmail.com (S.S.); 3 Department of Biology, College of Science, Imam Mohammad Ibn Saud Islamic University (IMSIU), Riyadh 11623, Saudi Arabia; thali@imamu.edu.sa (T.H.T.); saalsalamah@imamu.edu.sa (S.A.A.); 4 Biotechnology Program, Department of Mathematics and Natural Science, BRAC University, Dhaka 1212, Bangladesh; pranab.dev.sharma@g.bracu.ac.bd; 5 Department of Biology, College of Science, Qassim University, Buraydah 51452, Saudi Arabia

**Keywords:** viruses, vaccine, multi-epitope, immunoinformatics, in silico design, infectious disease

## Abstract

**Background:** Mammalian orthoreovirus is a ubiquitous double-stranded RNA virus that causes mild respiratory and enteric infections, primarily in infants and young children. Its significant environmental stability and association with conditions like celiac disease highlight an unmet medical need, as no licensed vaccine or antiviral treatment currently exist. **Methods:** An immunoinformatics-driven approach was employed to design a multi-epitope vaccine. The highly antigenic inner capsid protein Sigma-2 was used to predict cytotoxic T lymphocyte (CTL), helper T lymphocyte (HTL), and linear B cell epitopes using NetCTL, NetMHCpan, NetMHCIIpan, and IEDB tools. Selected epitopes were fused with appropriate linkers. The construct’s antigenicity, allergenicity, and physicochemical properties were evaluated. The tertiary structure was predicted with AlphaFold2, refined, and validated. Molecular docking with TLR2 and TLR4 was performed using HDOCK, and immune response simulation was conducted with C-ImmSim. Finally, the sequence was codon-optimized for *E. coli* expression using JCat. **Results:** The final vaccine construct comprises one CTL, four HTLs, and one B cell epitope. It is antigenic (VaxiJen score: 0.5026), non-allergenic, and non-toxic and possesses favorable physicochemical properties, including stability (instability index: 32.28). Molecular docking revealed exceptionally strong binding to key immune receptors, particularly TLR2 (docking score: −324.37 kcal/mol). Immune simulations predicted robust antibody production (elevated IgM, IgG1, and IgG2) and lasting memory cell formation. Codon optimization yielded an ideal CAI value of 0.952 and a GC content of 57.15%, confirming high potential for recombinant expression. **Conclusions:** This study presents a novel multi-epitope vaccine candidate against reovirus, designed to elicit broad cellular and humoral immunity. Comprehensive in silico analyses confirm its structural stability, potent interaction with innate immune receptors, and high potential for expression. These findings provide a strong rationale for further wet-lab studies to validate its efficacy and advance it as a promising prophylactic candidate.

## 1. Introduction

Reovirus is a double-stranded RNA virus, immersed in a 60–80 nm diameter nonenveloped icosahedral capsid from the *Reoviridae* family. Its genus name is orthoreovirus, in other words, “the true reovirus”. Due to its distinct nature, the virion exhibits enhanced stability against environmental conditions and physicochemical agents [1]. Reovirus causes mild or subclinical infections through respiratory and enteric routes. Because it was originally found in these systems without being linked to a specific disease, it was given the moniker “respiratory enteric orphan virus,” or reovirus. The food antigens of the mouth become intolerant because of this particular virus, which leads to celiac disease [2]. Celiac disease, such as autoimmune disorders with early-life exposure have been associated with gastrointestinal complications, suggesting that reovirus may play a broader role in human health than previously appreciated [3]. An experiment in a mouse model indicates that this virus can trigger a Th1 immunity response by upsetting homeostasis, which might have a connection to celiac disease, though other factors are also required. The infection caused by viruses possibly leaves a residue on the body, which later can activate autoimmune response towards gluten [4].

The airborne and fecal–oral routes are the methods of transmission to the host. A study showed that the virus can affect the upper respiratory tract with pharyngitis and rhinorrhea by showing symptoms of headache, fever, and malaise accompanied, or not, by minor diarrhea in children transmitted from adult volunteers [3,5]. However, it is possible that it will demonstrate threats in the future.

No treatment is available yet against reovirus [6]. Importantly, there are currently no licensed vaccines or antiviral therapies against reovirus, leaving susceptible populations—particularly infants and immunocompromised patients—without targeted protection [7]. This lack of preventive strategies points to the need for novel vaccine approaches.

The genus orthoreovirus is divided into two phylogenetic subgroups: fusogenic orthoreoviruses, which infect birds, reptiles, and mammals, and nonfusogenic orthoreoviruses, which specifically infect humans. Reovirus mainly targets mammals to make them hosts so they can spread infection. Their potential sites to cause disease are epithelial cells, and after that they spread to peripheral sites; they use both systemic routes and neural networks [1].

Traditional vaccine platforms, including live-attenuated and inactivated viruses, face challenges of biosafety, time-intensive development, and limited adaptability to emerging viral strains. Moreover, protein subunit vaccines require careful antigen selection and optimization to achieve adequate immunogenicity [8]. These barriers have motivated the adoption of computational vaccinology and immunoinformatics, which enable the rapid screening of viral proteomes, prediction of antigenic epitopes, and rational design of multi-epitope constructs with a reduced cost and development time [9].

Despite the success of immunoinformatics pipelines in the rational design of vaccines against pathogens such as SARS-CoV-2, Nipah virus, and Helicobacter pylori [10,11], no epitope-based vaccine has yet been reported for reovirus. Furthermore, while previous computational studies have predicted epitope assemblies for related viral pathogens, limited attention has been paid to host’s innate immune recognition, particularly the engagement of Toll-like receptors (TLRs) that orchestrate antiviral signaling. Addressing this gap, the present study applies an integrated in silico framework to design a multi-epitope vaccine construct from the reovirus Sigma-2 protein and evaluates its predicted structural stability, immunogenicity, and interactions with TLR2 and TLR4.

**Figure 1:**
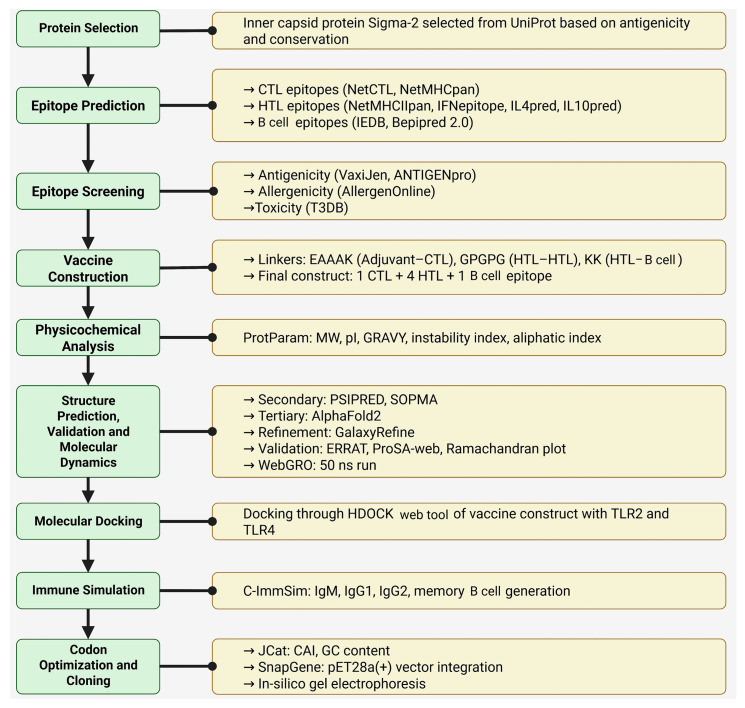
A diagrammatic representation of the in silico process.

This study aims to develop a vaccine by employing immunoinformatics methods to design a multi-epitope construct derived from a reovirus virulence protein, addressing the current lack of available therapeutics. This study uses immunoinformatics tools to identify epitopes, construct a multi-epitope vaccine, refine its structure, validate stability, perform molecular docking with TLR2/TLR4, simulate immune responses, and conduct in silico cloning, as there is no medication available. Traditional vaccine construction has drawbacks, such as being time-consuming and more expensive, which are good points for building vaccines by computational means [11]. A schematic representation of this study’s workflow is provided in Figure 1.

## 2. Results

### 2.1. Selection of CTL, HTL, and B Cell Epitopes

The NetMHCpanII 4.0 server was used to identify HTL epitopes that were strong binders. A total of 65 strong binder HTL epitopes were found after using this server. These HTL epitopes were further checked based on their IFN-γ, IL-4, and IL-10 stimulating profiles, which are shown in Table 1. The IFNepitope server was used to check IFN-γ. The IL-4pred server was used to the check IL-4 inducer, and IL-10pred server was used to check the IL-10 inducer. Sixteen HTL epitopes were maintained with all these criteria (Table 1).

The NetCTL1.2 server was used to identify the CTL epitopes, where this server uses the MHC-I supertype A1. A total of 12 CTL epitopes were found. The NetMHCpan-4.1 server was used to identify strong binding MHC-I alleles specific to CTL epitopes. Out of 12 CTL epitopes, 7 CTL epitopes were found that were specific to CTL epitopes. Then these CTL epitopes were further checked for antigenicity, allergenicity, and toxicity. Only one CTL epitope maintained all the criteria, and it was selected for vaccine construction. The antigenicity, allergenicity, and toxicity of the CTL epitopes are shown in the following table.

The sixteen HTL epitopes were further checked based on three parameters, which were antigenicity, allergenicity, and toxicity (Table 2). Six HTL epitopes were found that satisfy these criteria. Although six HTL epitopes satisfied all the screening criteria; they all are suitable for the construction of the vaccine. But among them, four were selected for the final construct based on their higher binding affinity, broader HLA allele coverage, and non-overlapping sequences, ensuring maximal immune diversity and construct stability.

Moreover, the B cell prediction tool IEDB Analysis Resource was used to determine the B cell epitopes, where different methods are available, but the Bepipred Linear Epitope Prediction 2.0 method was chosen as it is the most advanced method compared to the others. By using this server, a total of 10 epitopes were found and were further checked based on three parameters—antigenicity, allergenicity, and toxicity. After testing the B cell epitopes based on antigenicity, allergenicity, and toxicity stimulating profiles, it was found that only one B cell epitope satisfied all the criteria (Table 2) and was used for further constructing the vaccine.

### 2.2. Vaccine Construction

**Figure 2:**
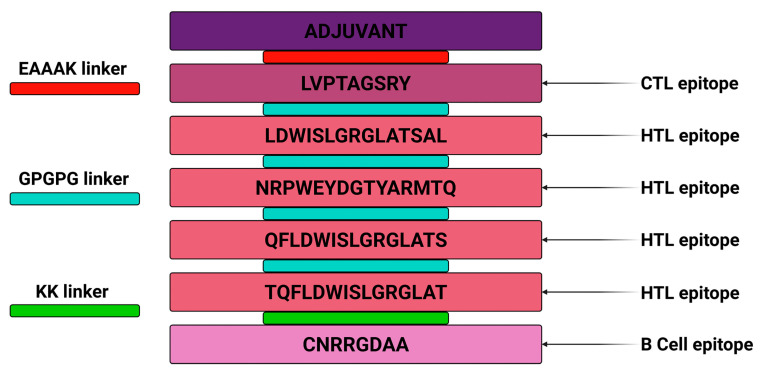
Multi-epitope vaccine construction through various linkers.

The final vaccine was assembled using the eligible epitopes and through linkers among them. First, the self-adjuvating scaffold or antigenic base was linked to the CTL epitope using the EAAAK linker, and here the protein sequence of the reovirus, which is “inner capsid protein Sigma-2” was used as the antigenic base or self-adjuvating scaffold because of its conserved, immunodominant, and antigenic ability to potentiate the immunogenicity of the linked epitopes. Next, the CTL epitope was linked to the HTL epitope using the GPGPG linker; then HTL epitopes were linked to HTL epitopes using the GPGPG linker, and, finally, the KK linker was used to connect HTL And B cell epitopes. The final vaccine was constructed by using one CTL, four HTLs, and one B cell epitope (Figure 2). 

### 2.3. Vaccine Physiochemical Properties

**Figure 3:**
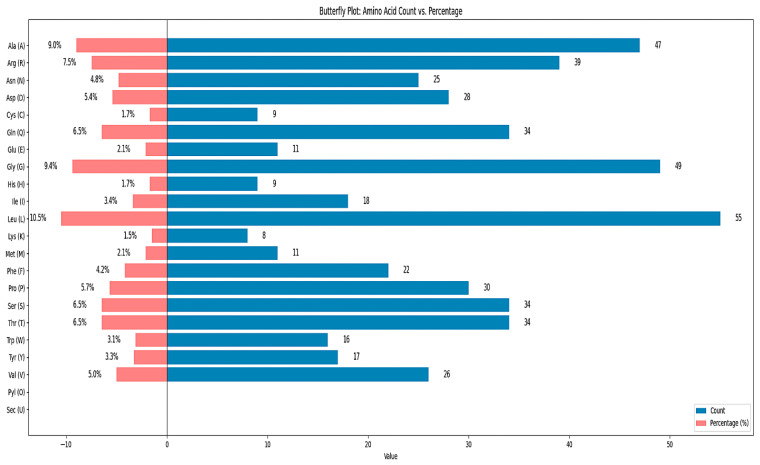
Amino acid count and percentage of designed multi-epitope vaccine.

The vaccine’s biochemical features contain attributes that may be verified using computational techniques, and an online tool called ProtParam is used to assess the physicochemical characteristics of the vaccine sequence (Table 3). Results of these properties, such as the molecular weight, amino acid number, therapeutic pI, GRAVY, instability index, aliphatic index, and other properties, are shown in the figure. The vaccine has a molecular weight of 57,869.50 Da with 522 amino acids (Figure 3 and Table 2). As the molecular weight and amino acid number are in the optimum range, it proves that the vaccine is stable. Additionally, the instability index is 32.28, and if it falls over 40, then the vaccine might have been unstable [12], which means this result falls within an acceptable range. Additionally, an aliphatic index value of 60 or above is always considered desirable. The grand average of hydropathicity (GRAVY) yielded a negative value, indicating that the vaccine is hydrophilic in nature [13]. Likewise, the 2D structure of the vaccine contains 20 helices, 6 beta strands, 15 beta turns, 3 gamma turns, and 2 beta hairpins.

### 2.4. Antigenicity, Allergenicity, and Toxicity of Vaccine

The Vaxijen v2.0 server was used to identify the vaccine’s antigenicity, and it displayed that the sequence was antigenic with a score of 0.5026 and a threshold of 0.5. ANTIGENpro (https://scratch.proteomics.ics.uci.edu/, accessed on 20 August 2025) online tool was used for the cross-validation of the antigenicity, and it showed that the antigenicity was 0.5297. The antigenicity was little bit higher, but it still provides a reliable indication of antigenicity, and marginally positive scores are considered biologically relevant. Additionally, to determine the allergenicity of the vaccine, the Allergen Online tool (http://www.allergenonline.org/, accessed on 20 August 2025) was used, and it showed that the hit number was one, which indicates the presence of an allergen in a very minute amount. Despite the fact that the in silico investigation suggested a small possibility of allergenicity, these predictions are conservative, and the structure’s logical design implies a substantial possibility of immunogenicity. In order to empirically confirm the safety and allergenicity of the substance before any clinical application, further research will be required. Furthermore, toxicity was assessed using the T3DB tool (https://www.t3db.ca/, accessed on 20 August 2025) to see whether any toxin particles or metabolites were present, and the result showed no toxicity.

### 2.5. Secondary and Tertiary Structure of Vaccine

#### 2.5.1. Predicted Secondary Structure

**Figure 4:**
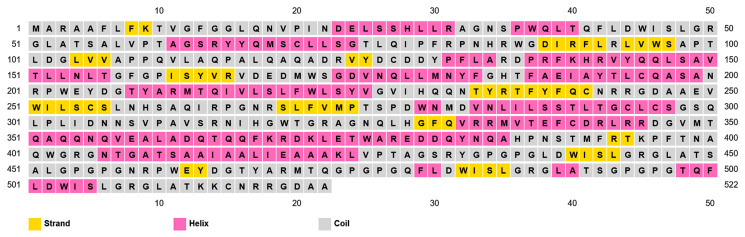
Predicted secondary structure of multi-epitope vaccine by PSIPRED.

**Figure 5:**
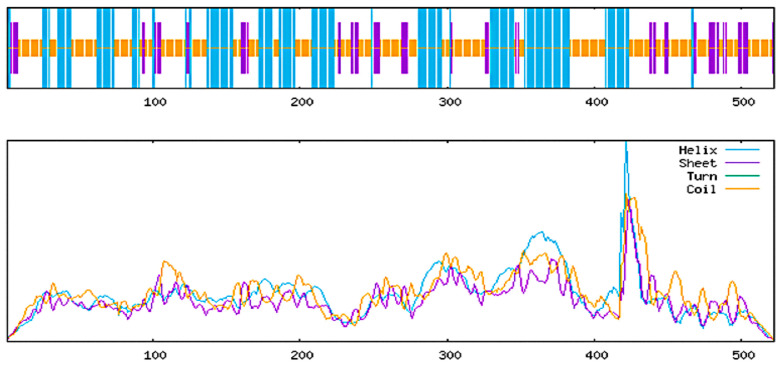
Predicted secondary structure of multi-epitope vaccine by SOPMA. In the top bar graph, different colors are used to show the predicted secondary structure types along a protein sequence of 500 amino acids. Blue bars represent helices, which are spiral-shaped regions; purple bars indicate sheets, which are flat and extended structures; and orange bars mark turns, which are short bends connecting other structural elements. In the bottom, line graph, the same structural types are shown using matching colors to represent their probability scores at each position in the sequence. A light blue line shows the likelihood of forming a helix, a green line shows the likelihood of forming a sheet, a purple line shows the likelihood of forming a turn, and an orange line shows the likelihood of forming a coil, which means no regular structure. These color-coded graphs help visualize both the type and confidence of predicted secondary structures, making it easier to understand the protein’s shape and stability.

The PSIPRED web server tool identified 36.5% alpha helixes, 10.9% strands, and 52.5% random coils (Figure 4). On the other hand, the SOPMA web server tool detected 34.7% alpha helixes, 12.8% extended strands, and 52.5% random coils from the vaccine sequence (Figure 5).

#### 2.5.2. Predicted Tertiary Structure

**Figure 6:**
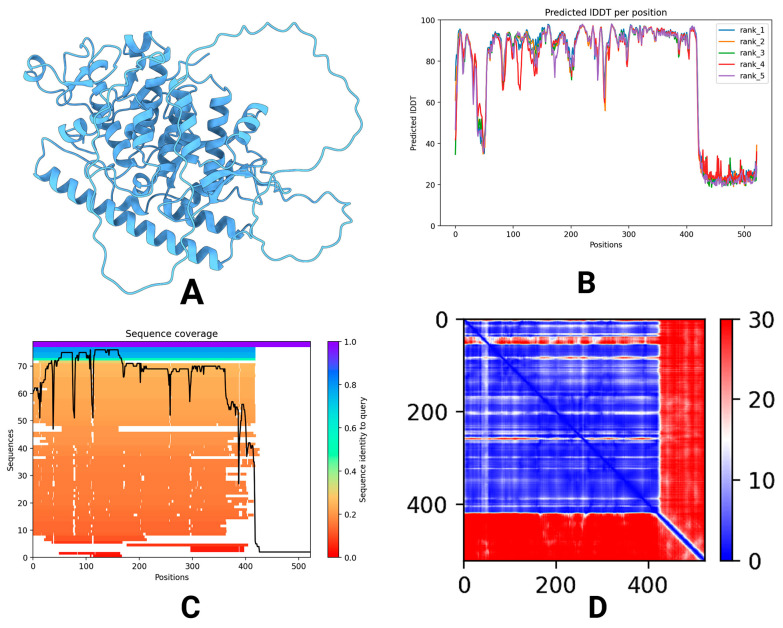
The tertiary structure prediction and model confidence assessment for the vaccine construct. (**A**) The predicted 3D structure of the vaccine. (**B**) Per-residue local confidence scores (pLDDT) for the five ranked models generated by AlphaFold2. (**C**) A Predicted Aligned Error (PAE) plot assessing the confidence in the relative position of residue pairs. (**D**) An inter-residue distance heatmap for the highest-confidence model (Model 3), with red indicating high prediction confidence.

The predicted tertiary structure of the vaccine design construct was modeled using AlphaFold2, and we received five ranked models (Figure 6A). Of those, Model 3 exhibited the best confidence in the structural inference (pLDDT of 76.5), whereby there were quite reliable residue-level predictions in the entire sequence (Figure 6B). Moreover, the predicted template modeling score (pTM) of 0.765 indicated a confident estimation on the global correctness of the fold (Figure 6C). The plDDT score measures the confidence in the predicted local structure of each protein residue, ranging from 0 to 100, with higher values indicating greater reliability and structural accuracy [14]. Likewise, the predicted template modeling score (pTM) assesses the degree of structural similarity between two folded protein models, ranging from 0 to 1. A pTM value above 0.5 suggests a significant resemblance, allowing for meaningful structural interpretations [15]. The visualization of Model 3 showed α-helices, β-strands, and loop regions that were spread across the entire construct, which corroborates its expected stability and its usability for immunological purposes (Figure 6D).

### 2.6. Structure Quality of Constructed Vaccine

**Figure 7:**
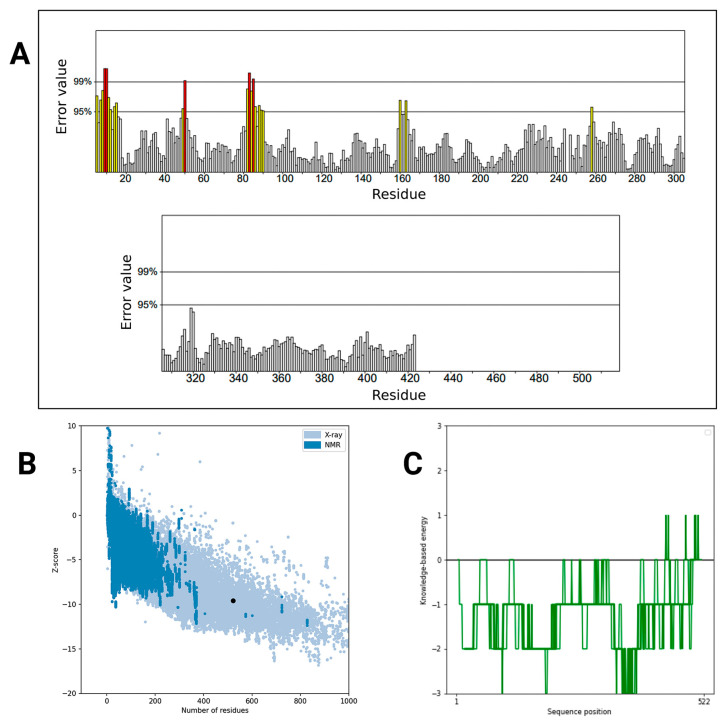
Protein structure validation and energy profiling. (**A**) Residue-level error analysis highlighting regions with values exceeding the 95% and 99% thresholds. Red bars indicate regions with high error values, suggesting structural instability or unreliable predictions, while yellow bars represent moderately high errors, possibly marking transitional zones. The remaining bars, shown in neutral tones, likely correspond to regions with low error values and stable predictions. (**B**) Scatter plot of Z-score versus number of residues comparing X-ray and NMR structural data. The black dot shows that the protein structure is within the X-ray data. Two shades of blue differentiate between experimental methods: dark blue points represent X-ray crystallography data, and light blue points correspond to NMR-derived structures. This color distinction helps visualize how Z-scores vary with the number of residues across methods. (**C**) The knowledge-based energy profile shows positional energy variations across the sequence. The green line shows knowledge-based energy values across the protein sequence. When the line goes below zero, it usually means the energy is favorable, suggesting that the structure in that region is likely stable or well-packed. When the line goes above zero, the energy is less favorable, which may point to unstable or strained regions in the protein.

The ERRAT plot evaluated the quality of the vaccine model, yielding a score of 94.724 (Figure 7A). This high score means that the majority of the protein domains are soundly constructed and not substantially misfolded. The number of positions where the error threshold was higher than 95% and 99% was minimal, suggesting satisfactory overall model accuracy. The structural validation by ProSA-web resulted in a Z-score of −9.59, which is a value typically observed for experimentally solved protein structures (Figure 7B). The more negative the value of the Z-score, the better the protein model. That is, the general shape and fold of the vaccine protein appear both stable and structurally sound. This knowledge-based energy profile showed the energy distribution along the protein sequence (Figure 7C). Very low energy values for the majority of regions were observed, suggesting well-folded and energetically favorable folding. Small peaks were noted in loop or flexible regions, as is typical for realistic biological representations.

**Figure 8:**
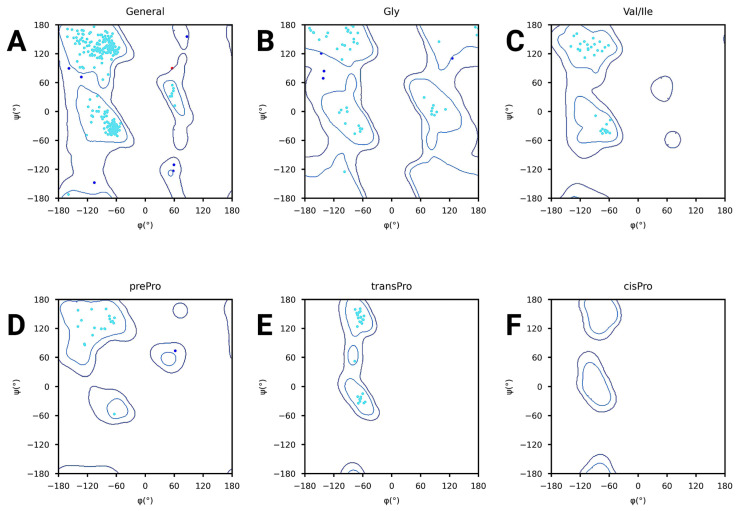
Ramachandran plots for vaccine structure validation. Light blue dots represent residues in favored regions, while red dots indicate outliers with unusual backbone angles. Dark blue dots often mark glycine residues due to their flexible conformations. (**A**) General distribution of backbone dihedral angles (φ and ψ) for all residues. (**B**) Glycine-specific plot showing flexible angle regions. (**C**) Valine and isoleucine residues highlighting restricted conformational zones. (**D**) Pre-Proline, (**E**) trans-Proline, and (**F**) cis-Proline plots displaying unique allowed regions based on residue type.

Furthermore, the Ramachandran plots reveal that the majority of the amino acid residues of the vaccine structure are located in the allowed regions, indicating that their angles are physically stable and have realistic values. The general plot of Figure 8A centers the majority of points in the favored regions, indicating good global geometry. For specific amino acids such as glycine in Figure 8B and proline types in Figure 8D–F, expected distributions were also obtained with only a few outliers. The valine and isoleucine plot in Figure 8C focuses on residues with more restricted conformational preferences. These values clearly indicate that the backbone angles (phi and psi) are reasonable for the model.

**Figure 9:**
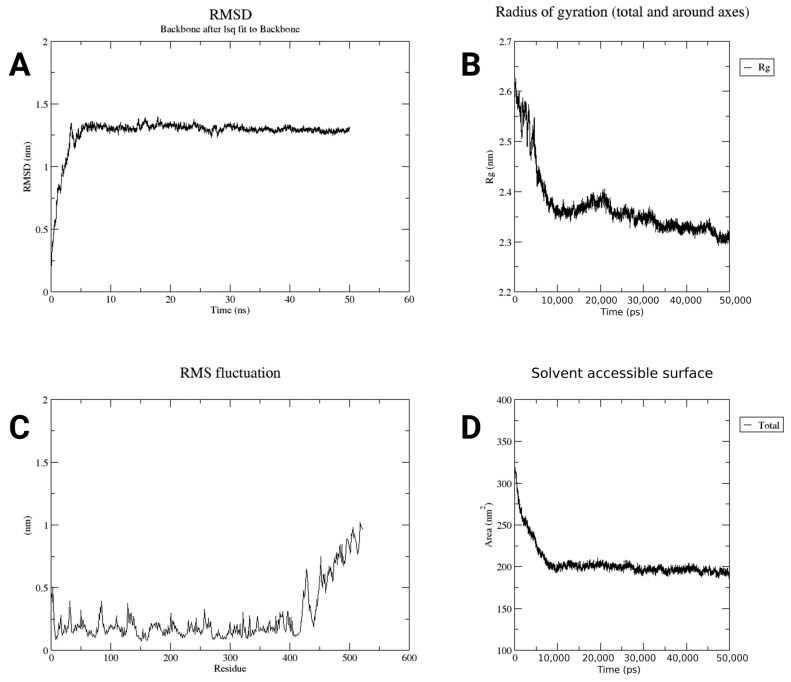
Molecular dynamics simulation analysis of the constructed vaccine. (**A**) RMSD plot showing structural stability of the protein backbone over time, indicating stabilization after ~10 ns. (**B**) Radius of gyration (Rg) analysis demonstrates that the protein becomes more compact during the simulation. (**C**) The RMSF plot represents the flexibility of individual residues. (**D**) Solvent-Accessible Surface Area (SASA) plot showing a decrease in exposed surface area, indicating proper folding and reduced water exposure.

Moreover, in the RMSD plot, the structure of the protein was quickly changed and became stable after 10 ns. The RMSD was kept between 1.3 and 1.5 nm, indicating little displacement of the structure after it had reached an equilibrium (Figure 9A). The gyration radius plot revealed that the protein was stretched but soon compacted and reached an equilibrium value of about 2.2 nm (Figure 9B). This result signified that the protein became more tightly folded and remained in that state for the rest of the simulation. As can be seen from the RMSF plot, almost the whole part of the protein does not move too much, and the fluctuation is below 0.5 nm. But the end part of the protein moved more, which is what is expected (Figure 9C). The ends of proteins are frequently looser and more dynamic. In the SASA graph, the protein’s surface area exposed to water was initially high (about 300 nm^2^), dropped fast, and became stable around 185 nm^2^ (Figure 9D). That is, the protein folded in such a way that it had little exposure to water. Taken together, these data indicate that the protein was stable and properly folded and behaved as expected during the simulation.

### 2.7. Simulation of Immune Response

**Figure 10:**
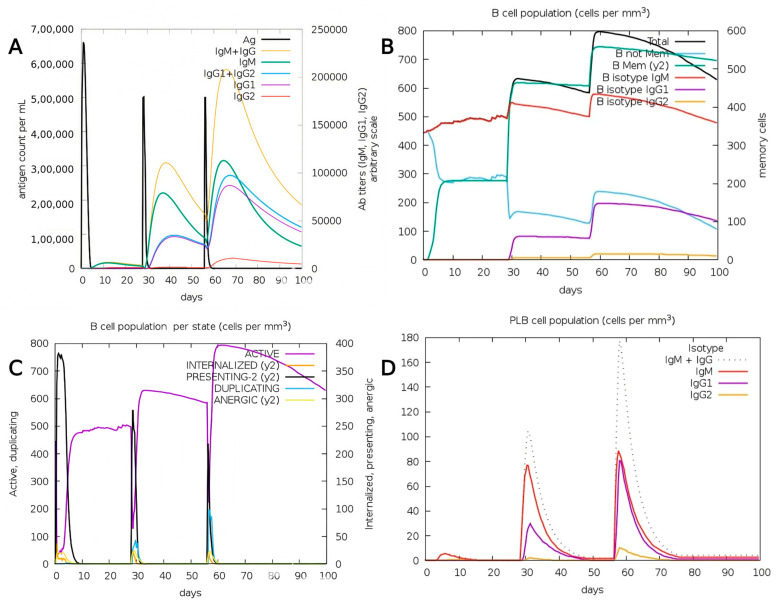
Immune response simulation analysis of the constructed vaccine. (**A**) Graphical re-presentation of antibody titers and antigen counts. (**B**) Graphical representation of the overall count of B lymphocytes, memory cells, and categorized immunoglobulins. (**C**) Graphical representation for B cell population per state. (**D**) Graphical representation of the plasma B lymphocyte cell population.

The C-ImmSim web browser is used for the immunological simulation to evaluate the immunoglobulin production capability of the developed vaccine in relation to dosage [16]. The server displayed various visual representations that elicited immunological responses. The antibody-producing capacity is demonstrated by the first figure following the administration of three doses of the vaccine. The black line on the graph represents the antigen, whereas the other colored lines denote the antibodies, as indicated in Figure 10A. The amount of antigen is at its peak on day 0, and there is no antibody. After injecting each dose, the antibody level gradually increased; in contrast, the antigen level fell in the second course. The rise in the antibody graphical line indicates an increase in our body’s ability to produce antibodies. The memory cell generation from B lymphocytes that retain a memory of the infection is shown in Figure 10B. Such production allows for the subsequent detection of the pathogen to identify it and initiate an immune response. The immune system is activated when the total B cell count rises after the antigen exposure. IgM-expressing B cells rise first, followed by increases in IgG1 and IgG2. A little slower growth in the memory B cell line indicates the development of long-term immunological memory. This pattern indicates a successful shift from a primary to a secondary humoral response, along with effective class switching and the establishment of long-term immunological memory. The noted rise in total and memory B cell populations, together with the consistent ratio of active and proliferating B cells, indicates the capacity of the modified structure to maintain the antigenic simulation and B cell differentiation. Figure 10C, which represents the B cell population entity state, shows that they are actively responding, as evidenced by the rise in the number of active or replicating B cells. The rise in both presenting and internalized B cells signifies their role in T cell presentation; nevertheless, there is also an increase in anergic B cells. Figure 10D shows the plasma B cell population with progressively increasing peaks of IgM, IgG1, and IgG2 antibodies. Plasma B cells are necessary for the maturation and release of antibodies in order for them to function in the immune system. Plasma B cells that are created by isotype IgM-producing plasma cells first increase, followed by IgG1- and IgG2-producing cells, which ultimately result in a decrease. The prevalence of IgG1 and IgG2 subclasses indicates a balanced Th1/Th2 immunological tendency. Th1-associated cytokines, such as IFN-γ, facilitate IgG2-type responses and cytotoxic T cell activation, whereas Th2 cytokines (IL-4 and IL-10) enhance IgG1 synthesis and antibody-mediated neutralization. The simultaneous occurrence of both characteristics indicates that the vaccine formulation may activate both cellular and humoral immunity, which is beneficial for extensive protection. However, it is important to point out that C-ImmSim is an in silico predictor, and the simulated antibody kinetics are modeled traits that necessitate experimental validation through in vitro and in vivo immunogenicity studies.

### 2.8. Docking Results

**Figure 11:**
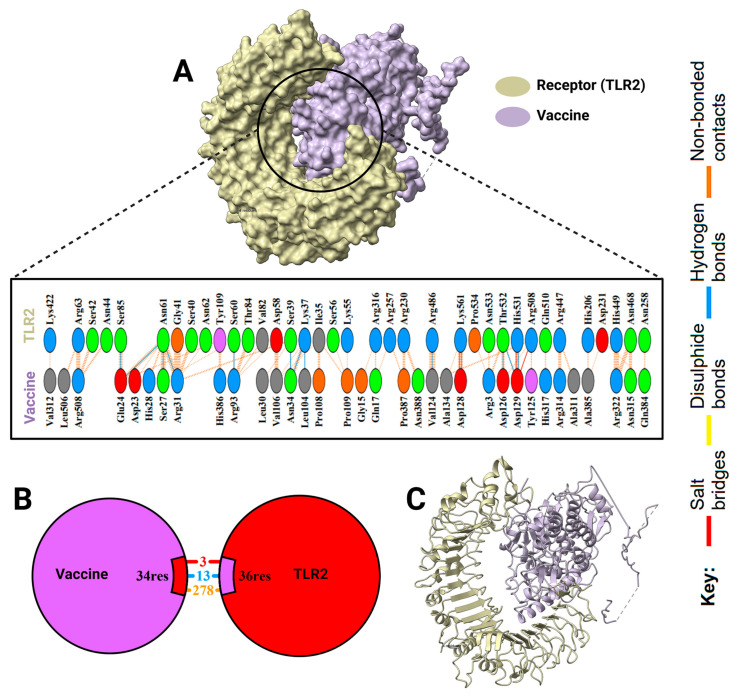
Analysis of the interaction of vaccine construct with TLR2. (**A**) The surface model of TLR2 in yellow and the vaccine in purple highlight the binding regions. (**B**) Zoom-in schematic representation of contact residues in binding, including 34 vaccine residues and 36 TLR2 residues, covering residues 3, 13, and 278. (**C**) A ribbon diagram illustrating the general conformation of the two molecules in docked poses. Different bond types, hydrogen bonds, salt bridges, disulfide bonds, and non-bonded interactions are indicated by a color-coded legend.

**Figure 12:**
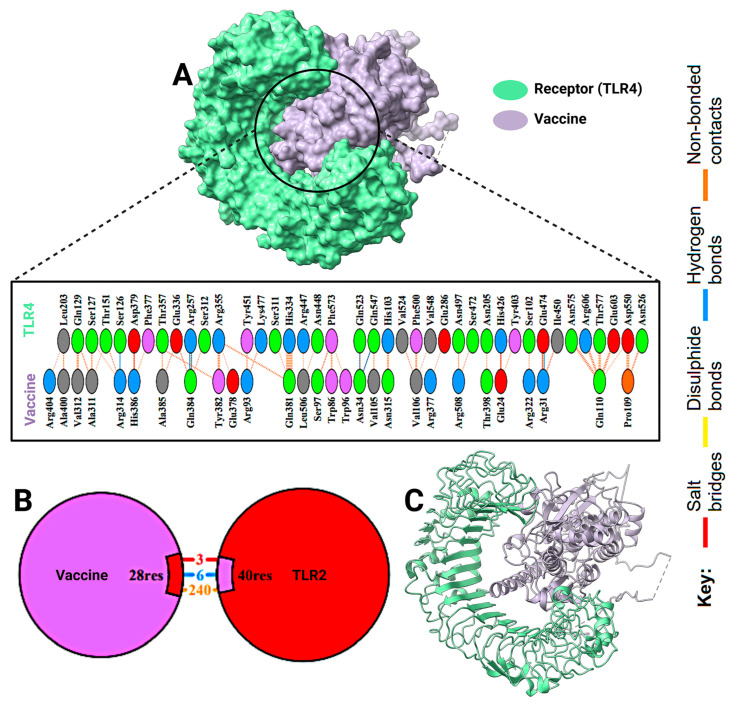
Analysis of the interaction of vaccine construct with TLR4. (**A**) The surface model of TLR4 in green and the vaccine in purple highlight the binding regions. (**B**) Zoom-in schematic representation of contact residues in binding, including 28 vaccine residues and 40 TLR4 residues, covering residues 3, 6, and 240. (**C**) A ribbon diagram illustrating the general conformation of the two molecules in docked poses. Different bond types, hydrogen bonds, salt bridges, disulfide bonds, and non-bonded interactions are indicated by a color-coded legend.

**Figure 13:**
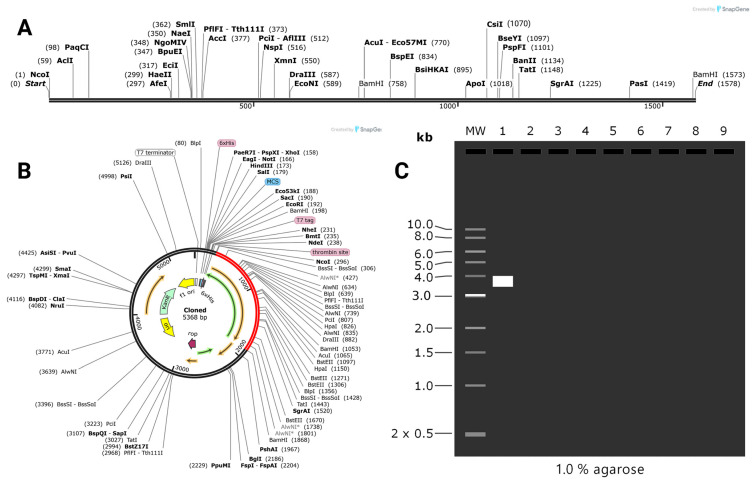
Cloning and vector integration of the vaccine construct. (**A**) A linear representation of the recombinant plasmid shows restriction sites and sequence features in base pairs. (**B**) Circular map of the pET28a(+) vector with the inserted vaccine gene shown in red. (**C**) Simulated agarose gel electrophoresis confirming successful insert ligation, where lane 2 displays a prominent band at approximately 3.0 kb.

A molecular docking study was executed to determine the binding affinity of the multi-epitope vaccine construct with Toll-like receptors (TLR2 and TLR4) (Figure 11 and Figure 12). The docking scores suggested strong interactions, but TLR2 had a higher binding score than TLR4 (−324.37 vs. −301.12, respectively). Corresponding confidence scores were 0.9703 for TLR2 and 0.9536 for TLR4, confirming the predicted complexes. A comprehensive interaction analysis showed high areas of contact between the vaccine and both receptors (Figure 13). The docked conformations displayed a variety of molecular interactions, including hydrogen bonds, salt bridges, disulfide bonds, and non-bonded forces. These data indicate stable, immunologically significant receptor–ligand complexes, which align with the construct’s capacity to elicit TLR-mediated immune responses.

### 2.9. Computational Cloning and Virtual Gel Analysis

To facilitate cloning and protein solubility production, the vaccine gene was codon-optimized for *E. coli* strain K12 expression using the Java Codon Adaptation Tool (JCat). The synthetic gene is 1565 nucleotides in size, has a codon adaptation index (CAI) of 0.952, and has a GC content of 57.15% (Figure 13A). These indices indicate that the gene may be expressed successfully in *E. coli*.

Following optimization, the gene sequence was ligated into the pET28a(+) expression vector based on the SnapGene 8.2.1 software. Scrolling from bottom to top through the SnapGene view, the vaccine sequence has been highlighted in red and the pET28a(+) vector in black (Figure 13B). The vaccine gene was inserted between the restriction sites NcoI (position 2986) and BamHI (position 1868) to create a full-length cloned construct of 5368 base pairs.

According to the in silico gel test, there is a clear band in the second lane, which is around 3.0 kilobase pairs. There were no other bands that appeared in lanes three to ten and no leftover DNA pieces, which means the vaccine gene was correctly added to the plasmid and working properly (Figure 13C).

### 2.10. Vaccine Coverage Worldwide

**Figure 14:**
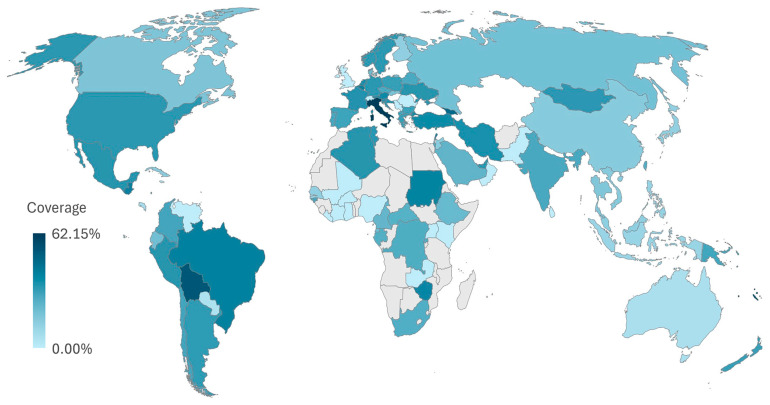
Global population coverage of selected vaccine epitopes based on MHC allele distribution. Coverage percentages were calculated using the IEDB Population Coverage tool (http://tools.iedb.org/population/, accessed on 20 August 2025) and are represented by country-specific shading.

The coverage was not the same in all countries. The highest coverage was found in Italy, with 62.15%. Some countries showed much lower values. A world map showing these differences is provided in Figure 14, where darker colors mean higher coverage. These results show that the selected epitopes may work well in many parts of the world. However, some regions may benefit more than others, depending on how common the matching alleles are. This highlights the need to include a wide range of HLA types when choosing epitopes for global vaccines.

## 3. Discussion

This study presents a novel multi-epitope vaccine candidate specifically targeting reovirus, a pathogen for which vaccine development has been historically neglected. Our integrated approach, combining rigorous epitope screening with advanced structural validation and immune simulation, provides a comprehensive blueprint for a potentially effective prophylactic measure. The strong predicted interaction with TLR2 and TLR4 is a particularly promising feature, as it suggests a capacity to robustly activate innate immunity, a critical first step in generating a potent adaptive response.

Despite a long history of research on its biology and epidemiology, effective vaccines against reovirus are unavailable. Despite typically mild or subclinical reovirus infections, these viruses have been implicated as contributors to the development of enteropathy, such as celiac disease, and early-life infections are proven to be preventable in animal models [4]. Conventional vaccine formats such as live attenuated or inactivated viruses have been effective against other pathogens but are labor-intensive, expensive, and potentially present biosafety hazards. Within this scheme of things, a immunoinformatics-driven multi-epitope vaccine design provides a rapid and cost-effective strategy by eliminating the excessive screening in the laboratory, allowing for the identification and preliminary selection of epitopes likely to be protective [17].

In the present study, we have identified one CTL, four HTLs, and one B cell epitope from the strongly antigenic inner capsid protein Sigma-2. These epitopes were rationally selected to ensure that the cellular and humoral immune responses would be optimized. The inclusion of CTL epitopes sustains the activation of cytotoxic T lymphocytes that are required for viral eradication [18,19], whereas HTL epitopes facilitate helper T cell activation and cytokine support [20], and B cell epitopes serve as targets for the generation of neutralizing antibodies [21,22]. It has been stressed in previous epitope vaccines that multi-tiered vaccination provides a synergistic activation of immune routes, which results in greater protection than a single epitope structure [23].

The physicochemical and structural characteristics of the vaccine candidate favor its stability and expression. The instability index score of the construct was less than 40, with a negative GRAVY value (hydrophilic and soluble protein). Such properties are particularly desirable for downstream processes and are often a prime handicap in the development of recombinant vaccine products [24]. The best structural model, along with the refined version and its validation using ERRAT and ProSA-web, was obtained for the predicted 3D structure of the vaccine through AlphaFold2. The results from the stereochemical check of the 3D structure suggested good folding and minimum errors. The results of the subsequent molecular dynamics simulations further supported this by showing structural stabilization during the 50 ns run, at which RMSD, radius of gyration, and SASA values were converged. Taken together, these results suggest that the construct may retain its structural integrity under physiological conditions, a requirement for predictably immunogenic efficacy.

The modulation of innate immune receptors is a potential molecular mechanism of the designed vaccine [25]. Docking studies revealed good binding affinities with TLR2 and TLR4, two pattern recognition receptors that are critically involved in molding antiviral immunity [26]. TLR2 has been linked to viral perception and pro-inflammatory signaling and TLR4 is known to activate pathways that enhance both innate and adaptive immunity with downstream signaling [27,28]. The high docking score and favorable complexes between the vaccine construct and these receptors obtained in this study indicate that the vaccine construct is capable of interacting with these receptors to trigger an effective immunological signaling cascade.

Computational immune simulations further supported the immunogenicity of the constructed vaccine. A modeled three-dose administration of the vaccine has shown high titers of IgM, IgG1, and IgG2 antibodies and was capable of memory B cell and T cell responses [10]. The reduction in the antigen concentration and the increase in the level of antibodies were consistent with what was expected in a successful vaccination strategy [29,30]. Importantly, the presence of long memory in the responses indicates that the vaccine construct may deliver long-lasting protection against reovirus infections. Although the predictions produced by C-ImmSim offer a theoretical approximation of immunological dynamics, the actual responses that occur in vivo may differ due to the complexity of the biological system. Consequently, these findings should be regarded as preliminary evidence necessitating additional experimental validation [31].

Another significant feature of this study is the codon optimization of the construct for heterologous expression. The codon adaptation for *E. coli* expression was high (0.952), and the GC content was moderate (57.15%), which is indicative of efficient transcription and translation in a prokaryotic organism [32]. This implies that the laboratory-scale test and production of the construct can be started, remaining cost-effective, and further experimental evaluation can be performed.

The findings are encouraging; however, their limitations need to be recognized. These are all predictions, and real immunogenicity, safety, and efficacy need to be confirmed experimentally. The in vivo presentation of epitopes is influenced by antigen processing and the host diversity of HLAs, which cannot be completely recapitulated by in silico tools. In addition, reovirus infections are age- and immune-status-dependent infections, and they should be validated in future animal models while taking into account these variables. One limitation of the current study is the use of *E. coli* as the expression system, which lacks the machinery for post-translational modifications such as glycosylation and proper protein folding. While codon optimization supports high expression, future experimental validation in eukaryotic systems may be necessary to ensure proper vaccine conformation and immunogenicity.

The present study represents a strong proof of concept for a multi-epitope reovirus vaccine. The combined antigenicity screening, structure-based refinement, molecular docking, immune simulation, and codon optimization render an integral pathway that could be used to design epitope-based vaccine constructs for other neglected or re-emerged viral pathogens. The combination of these computational methods decreases the time and cost associated with vaccine discovery and provides a selection of candidates that have the highest translational potential. To our knowledge, this study presents the first comprehensive design of a multi-epitope vaccine specifically targeting the reovirus Sigma-2 protein. The integrative strategy, which couples advanced structural prediction and validation with robust immune simulation, provides a novel blueprint for combating this neglected pathogen and demonstrates the ability of immunoinformatics to accelerate vaccine development for viruses that have eluded conventional approaches.

Despite these promising in silico results, this study has certain limitations. Immunoinformatics tools base their predictions on algorithms and existing datasets, which may not fully capture the complexity of in vivo immune responses. We still need to experimentally validate factors like host genetic diversity in HLA types, precise antigen processing and presentation in vivo, and potential off-target effects. Therefore, the findings presented here constitute a robust proof of concept that must be followed by in vitro and in vivo studies to confirm the immunogenicity, safety, and protective efficacy of the proposed vaccine candidate.

## 4. Materials and Methods

### 4.1. Protein Retrieval

For the selection of the protein sequence, the UniProt protein database (https://www.uniprot.org/, accessed on 20 August 2025) was used. From this server, different protein sequences were collected. This protein database contains 14,072 protein sequences for reovirus, where 242 sets are reviewed (Swiss-Prot), and 13,830 sets are unreviewed (TrEMBL). The antigenicity was checked to select the protein sequence, and that is why the protein sequences were downloaded in FASTA format. For checking the antigenicity, the Vaxijen v.2.0 (https://www.ddg-pharmfac.net/vaxijen/VaxiJen/VaxiJen.html, accessed on 20 August 2025) web server was used, where the targeted organism virus was selected, and the 0.5 threshold was maintained. The protein sequence was selected based on the highest score of antigenicity in viral replication for continued further steps, and the protein was an inner capsid protein Sigma-2. However, this protein was prioritized not only for its antigenicity but also for its structural and biological significance. It is a highly conserved core protein essential for capsid stability and viral replication, exhibiting minimal sequence variation across reovirus strains. Its partial surface exposure allows potential immune recognition, making it a strong vaccine target compared to other structural (e.g., λ1, μ1) or non-structural proteins that show higher variability or limited immunogenic potential. Thus, Sigma-2 was selected based on its high antigenicity, evolutionary conservation, and functional relevance for use in multi-epitope vaccine design.

### 4.2. Prediction and Evaluation of CTL Epitopes

For the prediction of CTL epitopes, NetCTL1.2 was used. CTL epitopes up to 12 histocompatibility complex class I subtypes (MHC-I) can be easily detected by using this server [33]. The protein sequence was provided in FASTA format, and a 0.75 threshold was maintained. NetMHCpan4.1 (https://services.healthtech.dtu.dk/services/NetMHCpan-4.1/, accessed on 20 August 2025) was used to evaluate the MHC-I binding alleles that are specific to CTL epitopes [34]. In this server, a 9mer peptide length was selected, and all the HLAs were selected. Thresholds of 0.5% and 2% were selected for strong and weak protein binding, respectively. The peptides fall within the range shown in the output page, and finally, the peptides that had strong protein binding were selected. These peptides were further checked for antigenicity, allergenicity, and toxicity.

### 4.3. Prediction and Evaluation of HTL Epitopes

NetMHCIIpan4.1 was used to predict HTL epitopes [34]. The protein sequence was provided in FASTA format, and 15 peptides were selected. A maximum of 20 alleles were selected per submission. Thresholds of 1% and 5% were selected for strong and weak binders, respectively. The result page showed the alleles that fell within this threshold, and alleles that had high binding affinity were selected. For the evaluation of whether HTL epitopes were IFN-gamma inducers or not, the IFNepitope (http://crdd.osdd.net/raghava/ifnepitope/, accessed on 20 August 2025) web server was used [35]. Again, these HTL epitopes were evaluated to check whether they were IL-4 inducers or non-inducers and IL-10 inducers or non-inducers, IL-4pred (http://crdd.osdd.net/raghava/il4pred/, accessed on 20 August 2025) and IL-10pred (https://webs.iiitd.edu.in/raghava/il10pred/predict3.php, accessed on 20 August 2025) servers were used, respectively [36,37]. HTL epitopes that maintained all the criteria were further checked for antigenicity, allergenicity, and toxicity.

### 4.4. Prediction and Evaluation of B Cell Epitopes

IEDB Resource Analysis Server (http://tools.iedb.org/main/, accessed on 20 August 2025) was used to predict B cell epitopes [38]. The protein sequence was presented in plain format, and a threshold of 0.5 was maintained. The result page showed a graph and a table based on antigenicity. Those peptides that fell within the threshold range were selected and further checked for antigenicity, allergenicity, and toxicity.

### 4.5. Constructed Vaccined

A multi-epitope vaccine was created by combining all the chosen epitopes, which were CTL, HTL, and B cell sequences, and linkers were used to separate each individual sequence [39]. The choice of appropriate linkers is very important because a lack of linkers can cause peptide-based vaccination misfolding, which further reduces vaccines’ biological activity [8]. The most common linkers utilized to unite CTL, HTL, and B cell epitopes are AAY, GPGPG, and KK. Linker EAAAK was used to link adjuvant and CTL epitopes.

### 4.6. Structural and Chemical Attributes

The physical and chemical characteristics of proteins are essential for sustainability, efficiency, and stability within an organism [40]. The hypothetical protein’s simple amino acid sequence was analyzed using ExPASy’s ProtParam (https://web.expasy.org/protparam/, accessed on 20 August 2025) tool to determine its theoretical physiochemical properties [41]. Several physico-chemical characteristics, including atomic composition, molecular weight, instability index, GRAVY, aliphatic index, and theoretical pI, facilitate the comprehension of their stability, activity, and nature. For identifying proteins based on their amino acid composition, ExPASy has developed an online application called AAComplement. The grand average of hydropathy is the hydropathy value calculated by dividing the number of residues by the sum of all amino acids. A positive GRAVY score denotes hydrophobicity, while a negative number suggests hydrophilicity [40].

### 4.7. Antigenicity and Allergenicity of Vaccine

Antigenicity, allergenicity, and toxicity are other important parameters that need to be checked to ensure vaccine safety and efficacy. To eliminate the potential for allergic or toxic properties of the proposed vaccine, it undergoes additional screening using online allergenicity prediction tools like AllergenOnline and toxicity prediction tools like T3DB (T3DB: The Toxic Exposome Database. Wishart et al., University of Alberta, Canada. Available at: https://www.t3db.ca, accessed on 20 August 2025). Using VaxiJen v2.0 (https://www.ddg-pharmfac.net/vaxijen/VaxiJen/VaxiJen.html, accessed on 20 August 2025), the vaccine sequence may be further examined for its antigenicity once allergenicity and toxicity have been eliminated [9]. However, AntigenPro (https://scratch.proteomics.ics.uci.edu/, accessed on 20 August 2025) was used to crosscheck the antigenicity.

### 4.8. Secondary and Tertiary Structure Prediction of Vaccine

#### 4.8.1. Secondary Structure

Widely used computational tools were used to analyze the secondary structure of the engineered vaccine construct. The latter secondary structure was first predicted with PSIPRED (https://bioinf.cs.ucl.ac.uk/psipred/, accessed on 22 August 2025), which employs PSI-BLAST and a neural network method to locate α-helices, β-strands, and coils. It provides precise visual and numerical information with regard to evolutionary information [42]. Structural elements were sorted, applying two-dimensional alignment of sequences; analysis of frequency; and determinant prediction of α-helices, β-sheets, turns, and coils with SOPMA (https://npsa.lyon.inserm.fr/cgi-bin/npsa_automat.pl?page=/NPSA/npsa_sopma.html, accessed on 22 August 2025). This is a visual representation of the proportions of all structure types with changeable options [43]. These integrated tools further increase the confidence of the results obtained by comparative modeling. They pinpoint essential structural regions involved in the protein’s folding, stability, and immune responses.

#### 4.8.2. Tertiary Structure Prediction

The structure of the vaccine was modeled with the AlphaFold2 Collab platform, a cutting-edge deep learning framework for protein structure prediction (https://colab.research.google.com/github/sokrypton/ColabFold/blob/main/AlphaFold2.ipynb#scrollTo=AzIKiDiCaHAn, accessed on 22 August 2025). AlphaFold2 Collab uses a multistep approach with several neural network architectures [44]. First, the system predicts distances and orientations between residues and carries out a refinement step that uses other structural information, leading to better results [45]. This server facilitates rapid and accurate protein structure prediction using state-of-the-art deep learning methods and large training datasets [46]. This results in the production of highly accurate protein models, ready for further analysis and interpretation.

### 4.9. Structure Refiner

The 3D model of the constructed multi-epitope vaccine was optimized with GalaxyRefine (https://galaxy.seoklab.org/cgi-bin/submit.cgi?type=REFINE, accessed on 22 August 2025), a computationally expensive application resource available on the web from the Seok Lab of Seoul National University. GalaxyRefine improves protein models by iteratively repacking the side chains and subjecting residues and the model to short molecular dynamics (MD) simulations for structure relaxation [47]. This approach has been successfully tested in CASP experiments and shows some ability to recover the local and global structures [47,48]. For the input PDB model, the server produced five refined models. The best model was chosen by the following quality criteria: GDT-HA, RMSD, MolProbity score, Ramachandran plot statistics, and better stereochemical quality and backbone geometry [47].

### 4.10. Structure Quality of Vaccine

#### 4.10.1. Errat

ERRAT was used to verify the prediction precision as well as the reliability of predicted protein models (https://saves.mbi.ucla.edu/, accessed on 22 August 2025). ERRAT is a web program for verifying protein structures by inspecting non-bonded atoms. It takes the anticipated atom–atom contact patterns in the model and compares them with observed ones to locate structural discrepancies [49]. People generally regard models with an overall value higher than 90% as having good structural quality and accuracy [50].

#### 4.10.2. ProSa-Web

Additional verification was carried out by ProSa-Web (https://prosa.services.came.sbg.ac.at/prosa.php, accessed on 22 August 2025), a web-based application that allows the detection of possible errors in three-dimensional protein models [51]. ProSa-Web provides a Z-score whether or not the model fits the experimentally solved models. The more negative the Z-score, the better and more stable the protein structure. There are also energy plots from ProSa-Web that indicate problematic parts of the structure. Energy plots can be employed to visualize local structural problems [52,53].

#### 4.10.3. Ramachandran Plotting

The structural geometry was also examined in the Ramachandran plot of the model by Ramplot (https://ramplot.in/index.php, accessed on 23 August 2025), where phi (φ) and psi (ψ) torsional angles of amino acids of the protein backbone were displayed [54]. On the plot, the x-axis is the φ angles, and the y-axis is the ψ angles. Favorable conformations universally found in proteins are indicated. These areas are the right-handed alpha helix, left-handed alpha helix, and beta-sheet regions. The large proportion of amino acids located in these favorable zones evidences superior folding and structure stability.

#### 4.10.4. Vaccine in Water Simulation

To understand how the vaccine behaves in water, a molecular dynamics simulation was carried out using the WebGRO (WebGRO for Macromolecular Simulations. University of Arkansas for Medical Sciences, USA. Available at: https://simlab.uams.edu, accessed on 23 August 2025) online tool [55]. First, the vaccine’s 3D structure (PDB file) was uploaded, and the GROMOS96 54a7 force field was chosen to define how atoms interact. The simulation environment was set up with the SPC water model and enclosed within a triclinic box. To mimic physiological conditions, the system was neutralized and supplemented with 0.15 M salt. Energy minimization was performed using the steepest descent method for 10,000 steps, helping eliminate unfavorable contacts and stabilize the structure. Next, the system underwent equilibration in both NVT (constant number of particles, volume, and temperature) and NPT (constant number of particles, pressure, and temperature) ensembles, maintaining a temperature of 298 K and a pressure of 1.5 bar. Following equilibration, the production MD run was initiated using the leapfrog integrator. The simulation ran for 50 nanoseconds, and 5000 frames were generated to capture detailed motion and interaction patterns of the vaccine in the water environment.

### 4.11. Immune Response Simulation

To better understand the immune system, computational techniques have become more advanced. Immune simulation was performed using the C-ImmSim (https://kraken.iac.rm.cnr.it/C-IMMSIM/index.php, accessed on 25 August 2025) server to confirm the immune response of the proposed vaccine [35]. The immunogenicity of the vaccine in terms of its amino acid sequence may be tested using the immune system simulation server to test whether it could activate immune system cells [56]. The total number of simulation steps was 1050, with three injections administered every week. The time-step parameters were set at 1, 84, and 168, with one time-step representing 8 h in real life [18].

### 4.12. Molecular Docking

#### 4.12.1. Protein Preparation

Three Toll-like receptors (TLRs), such as TLR2 (PDB ID: 6NIG) and TLR4 (PDB ID: 3FXI), were used for docking with the constructed vaccine. The 3D structures of those proteins were downloaded from the Protein Data Bank (https://www.rcsb.org/, accessed on 25 August 2025). The vaccine structure was saved in PDB format. All protein files were cleaned using UCSF Chimera X1.9 to remove water molecules, unwanted atoms, and alternate shapes [57].

#### 4.12.2. Docking Setup

Docking was performed using the HDOCK server (http://hdock.phys.hust.edu.cn/, accessed on 25 August 2025) [58]. In each run, one TLR protein was uploaded as the “receptor”, and the vaccine was uploaded as the “ligand.” The standard settings were used without any extra restraints. No specific binding site was set, allowing the program to scan the full surface [59]. After submitting the job, HDOCK ran the docking using a hybrid method that combines template-based and free docking. The results showed several possible complexes, ranked by score. The top-ranked model was selected for further analysis based on docking score and interaction pattern, which included hydrogen bonds and hydrophobic contacts.

#### 4.12.3. Model Analysis and Visualization

After docking, the best models with low energy scores and large cluster sizes were picked for further study. These structures were viewed using UCSF Chimera X1.9 and the PDBsum web server (https://www.ebi.ac.uk/thornton-srv/databases/pdbsum/, accessed on 26 August 2025) [57,60]. The interactions between the vaccine and TLRs were examined, including hydrogen bonds, hydrophobic areas, and salt bridges. The binding areas were studied and compared across TLR2 and TLR4 to understand how well the vaccine might trigger an immune response.

### 4.13. In Silico Cloning and Gel Electrophoresis

To enhance the efficacy of the multi-epitope vaccine, the gene sequence was optimized using the Java Codon Adaptation Tool (JCat) (JCat: Java Codon Adaptation Tool. Grote et al., Braunschweig University of Technology, Germany. Available at: https://www.jcat.de, accessed on 26 August 2025). This process enabled codon adaptation for the vaccine to effectively express in *E. coli* strain K12. Some important decisions were made to cause the improved vector to minimize potential disruption of gene expression, such as avoiding rho-independent transcription terminators, excluding prokaryotic RBS (ribosome binding sites), and restriction sites that could interfere in cloning. JCat presents you two important outputs, namely the codon adaptation index (CAI) and the %GC of the synthesized sequence. The ideal GC content is from 30% to 70% for satisfactory translation, expression stability, and strong transcription [61]. he CAI value should be more than 0.8 or approach 1.0, which indicates a high expression of the gene [62]. The improved sequence was cloned using the SanpGene 3.2.1 software. Moreover, the in silico gel electrophoresis was run by the SnapGene software for validating the cloning of the vaccine construct, available at https://www.snapgene.com/, accessed on 26 August 2025.

### 4.14. Population Coverage

To identify populations most likely to benefit from the selected vaccine epitopes, population coverage was predicted using the Population Coverage tool available on the IEDB server (http://tools.iedb.org/population/, accessed on 20 August 2025). Finalized MHC class I and class II allele data were submitted to estimate coverage across global population groups [63].

## 5. Conclusions

This study provides a rational immunoinformatics-driven design of a multi-epitope vaccine candidate targeting the highly antigenic Sigma-2 protein of reovirus. By integrating CTL, HTL, and B cell epitopes with optimized linkers and an adjuvant, the construct demonstrated favorable physicochemical properties, structural stability, and strong in silico binding affinity with innate immune receptors TLR2 and TLR4. Computational immune simulations further predicted robust humoral and cellular responses, while codon optimization supported potential expression in a heterologous host. We must emphasize that all results are based on computational predictions, despite these encouraging findings. We still need to establish the true immunogenicity, safety, and protective efficacy of the construct, both in vitro and in vivo. Translational gaps, including host-specific antigen processing, population-level HLA diversity, and the complexity of immune regulation, represent critical hurdles before clinical applicability can be envisioned. Nonetheless, this work highlights the power of immunoinformatics pipelines as a first step in accelerating vaccine discovery for pathogens such as reovirus, where no licensed vaccines currently exist. By reducing the time and cost compared to empirical screening, such approaches can rapidly generate rational vaccine candidates and guide experimental prioritization. Future validation in laboratory and animal models will be essential to confirm the protective potential of this construct and to advance it toward a viable prophylactic strategy against reovirus infections.

## Figures and Tables

**Table 1:** Predicted HTL epitopes with confirmed IFN-γ, IL-4, and IL-10 stimulation profiles.

HTL Epitope	IFN	IL4	IL10
ANRPWEYDGTYARMT	Positive	Inducer	Inducer
AVSRNIHGWTGRAGN	Positive	Inducer	Inducer
DDYPFLARDPRFKHR	Positive	Inducer	Inducer
DYPFLARDPRFKHRV	Positive	Inducer	Inducer
FKHRVYQQLSAVTLL	Positive	Inducer	Inducer
FLDWISLGRGLATSA	Positive	Inducer	Inducer
LDWISLGRGLATSAL	Positive	Inducer	Inducer
NRPWEYDGTYARMTQ	Positive	Inducer	Inducer
PFLARDPRFKHRVYQ	Positive	Inducer	Inducer
QFLDWISLGRGLATS	Positive	Inducer	Inducer
RPWEYDGTYARMTQI	Positive	Inducer	Inducer
SANRPWEYDGTYARM	Positive	Inducer	Inducer
SRNIHGWTGRAGNQL	Positive	Inducer	Inducer
TQFLDWISLGRGLAT	Positive	Inducer	Inducer
VSRNIHGWTGRAGNQ	Positive	Inducer	Inducer
YPFLARDPRFKHRVY	Positive	Inducer	Inducer

**Table 2:** Predicted CTL, HTL, and B cell epitopes with confirmed antigenicity, non-allergenicity, and non-toxicity profiles.

Epitope Type	Epitope Sequence
**CTL epitope**	LVPTAGSRY
**HTL epitope**	ANRPWEYDGTYARMT FLDWISLGRGLATSA LDWISLGRGLATSAL NRPWEYDGTYARMTQ QFLDWISLGRGLATS TQFLDWISLGRGLAT
**B cell epitope**	CNRRGDAA

**Table 3:** Physiochemical properties of the constructed vaccine sequence.

Properties	Value
Total carbon	2577
Total hydrogen	3960
Total nitrogen	740
Total oxygen	745
Total sulfur	20
Total number of atoms	8042
Formula	C_2577_H_3960_N_740_O_745_S_20_
Estimated half-life	30 h (mammalian reticulocytes, in vitro) >20 h (yeast, in vivo) >10 h (*Escherichia coli*, in vivo)
Instability index	32.28 (protein is stable)
Aliphatic index	77.99
Grand average of hydropathicity (GRAVY)	−0.277
Number of amino acids	522
Theoretical pI	9.02
Molecular weight	57,869.50

## Data Availability

Data are contained within the article.

## References

[1] Betancourt W.Q., Gerba C.P. (2016). Rethinking the Significance of Reovirus in Water and Wastewater. Food Environ. Virol..

[2] Eledge M.R., Zita M.D., Boehme K.W. (2019). Reovirus: Friend and Foe. Curr. Clin. Microbiol. Rep..

[3] Asri N., Mohammadi S., Jahdkaran M., Rostami-Nejad M., Rezaei-Tavirani M., Mohebbi S.R. (2025). Viral infections in celiac disease: What should be considered for better management. Clin. Exp. Med..

[4] Bouziat R., Hinterleitner R., Brown J.J., Stencel-Baerenwald J.E., Ikizler M., Mayassi T., Meisel M., Kim S.M., Discepolo V., Pruijssers A.J. (2017). Reovirus infection triggers inflammatory responses to dietary antigens and development of celiac disease. Science (1979).

[5] Rosen L., Evans H.E., Spickard A. (1963). REOVIRUS INFECTIONS IN HUMAN VOLUNTEERS. Am. J. Epidemiol..

[6] DeBiasi R.L., Tyler K.L. (2015). Orthoreoviruses and Orbiviruses. Mandell, Douglas, and Bennett’s Principles and Practice of Infectious Diseases.

[7] Pan M., Alvarez-Cabrera A.L., Kang J.S., Wang L., Fan C., Zhou Z.H. (2021). Asymmetric reconstruction of mammalian reovirus reveals interactions among RNA, transcriptional factor µ2 and capsid proteins. Nat. Commun..

[8] Gong W., Pan C., Cheng P., Wang J., Zhao G., Wu X. (2022). Peptide-Based Vaccines for Tuberculosis. Front. Immunol..

[9] Kuri P.R., Goswami P. (2022). Current Update on Rotavirus in-Silico Multiepitope Vaccine Design. ACS Omega.

[10] Sharma S., Yadav P.D., Cherian S. (2025). Comprehensive immunoinformatics and bioinformatics strategies for designing a multi-epitope based vaccine targeting structural proteins of Nipah virus. Front. Immunol..

[11] Ma J., Qiu J., Wang S., Ji Q., Xu D., Wang H., Wu Z., Liu Q. (2021). A Novel Design of Multi-epitope Vaccine Against Helicobacter pylori by Immunoinformatics Approach. Int. J. Pept. Res. Ther..

[12] Guruprasad K., Reddy B.V.B., Pandit M.W. (1990). Correlation between stability of a protein and its dipeptide composition: A novel approach for predicting in vivo stability of a protein from its primary sequence. Protein Eng..

[13] Wang H., Zhong H., Gao C., Zang J., Yang D. (2021). The Distinct Properties of the Consecutive Disordered Regions Inside or Outside Protein Domains and Their Functional Significance. Int. J. Mol. Sci..

[14] Carugo O. (2023). pLDDT Values in AlphaFold2 Protein Models Are Unrelated to Globular Protein Local Flexibility. Crystals.

[15] Wuyun Q., Chen Y., Shen Y., Cao Y., Hu G., Cui W., Gao J., Zheng W. (2024). Recent Progress of Protein Tertiary Structure Prediction. Molecules.

[16] Asif Rasheed M., Awais M., Aldhahrani A., Althobaiti F., Alhazmi A., Sattar S., Afzal U., Ali Baeshen H., Ali El Enshasy H., Joe Dailin D. (2021). Designing a highly immunogenic multi epitope based subunit vaccine against Bacillus cereus. Saudi J. Biol. Sci..

[17] Kothiyal P., Pant K., Singh P. (2025). Immunoinformatics-Based Vaccine Designing. Advancing Biotechnology: From Science to Therapeutics and Informatics.

[18] Andersen M.H., Schrama D., Thor Straten P., Becker J.C. (2006). Cytotoxic T Cells. J. Investig. Dermatol..

[19] Huber S.R., van Beek J., de Jonge J., Luytjes W., van Baarle D. (2014). T cell responses to viral infections-opportunities for peptide vaccination. Front. Immunol..

[20] Alexander J., Fikes J., Hoffman S., Franke E., Sacci J., Appella E., Chisari F.V., Guidotti L.G., Chesnut R.W., Livingston B. (1998). The optimization of helper T lymphocyte (HTL) function in vaccine development. Immunol. Res..

[21] Sanchez-Trincado J.L., Gomez-Perosanz M., Reche P.A. (2017). Fundamentals and Methods for T- and B-Cell Epitope Prediction. J. Immunol. Res..

[22] Noorimotlagh Z., Karami C., Mirzaee S.A., Kaffashian M., Mami S., Azizi M. (2020). Immune and bioinformatics identification of T cell and B cell epitopes in the protein structure of SARS-CoV-2: A systematic review. Int. Immunopharmacol..

[23] Invenção M.d.C.V., Macêdo L.S.d., Moura I.A.d., Santos L.A.B.d.O., Espinoza B.C.F., Pinho S.S.d., Leal L.R.S., Santos D.L.d., São Marcos B.d.F., Elsztein C. (2025). Design and Immune Profile of Multi-Epitope Synthetic Antigen Vaccine Against SARS-CoV-2: An In Silico and In Vivo Approach. Vaccines.

[24] Gul S., Ahmad S., Ullah A., Ismail S., Khurram M., Qamar M.T.U., Hakami A.R., Alkhathami A.G., Alrumaihi F., Allemailem K.S. (2022). Designing a Recombinant Vaccine against Providencia rettgeri Using Immunoinformatics Approach. Vaccines.

[25] Samantaray M., Pushan S.S., Rajagopalan M., Abrol K., Basumatari J., Murthy T.P.K., Ramaswamy A. (2025). Designing a multi-epitope vaccine candidate against pandemic influenza a virus: An immunoinformatics and structural vaccinology approach. Mol. Divers..

[26] Lester S.N., Li K. (2014). Toll-Like Receptors in Antiviral Innate Immunity. J. Mol. Biol..

[27] Oliveira-Nascimento L., Massari P., Wetzler L.M. (2012). The role of TLR2 ininfection and immunity. Front. Immunol..

[28] Kim H.J., Kim H., Lee J.H., Hwangbo C. (2023). Toll-like receptor 4 (TLR4): New insight immune and aging. Immun. Ageing.

[29] Wei J., Zhang Y., Li H., Wang F., Yao S. (2023). Toll-like receptor 4: A potential therapeutic target for multiple human diseases. Biomed. Pharmacother..

[30] Mohammadipour S., Tavakkoli H., Fatemi S.N., Sharifi A., Mahmoudi P. (2025). Designing a multi-epitope universal vaccine for concurrent infections of SARS-CoV-2 and influenza viruses using an immunoinformatics approach. BMC Infect. Dis..

[31] Tahir ul Qamar M., Rehman A., Tusleem K., Ashfaq U.A., Qasim M., Zhu X., Fatima I., Shahid F., Chen L.-L. (2020). Designing of a next generation multiepitope based vaccine (MEV) against SARS-COV-2: Immunoinformatics and in silico approaches. PLoS ONE.

[32] Jain R., Jain A., Mauro E., LeShane K., Densmore D. (2023). ICOR: Improving codon optimization with recurrent neural networks. BMC Bioinform..

[33] Larsen M.V., Lundegaard C., Lamberth K., Buus S., Lund O., Nielsen M. (2007). Large-scale validation of methods for cytotoxic T-lymphocyte epitope prediction. BMC Bioinform..

[34] Reynisson B., Alvarez B., Paul S., Peters B., Nielsen M. (2020). NetMHCpan-4.1 and NetMHCIIpan-4.0: Improved predictions of MHC antigen presentation by concurrent motif deconvolution and integration of MS MHC eluted ligand data. Nucleic Acids Res..

[35] Kupani M., Pandey R.K., Vashisht S., Singh S., Prajapati V.K., Mehrotra S. (2023). Prediction of an immunogenic peptide ensemble and multi-subunit vaccine for Visceral leishmaniasis using bioinformatics approaches. Heliyon.

[36] Dhanda S.K., Gupta S., Vir P., Raghava G.P. (2013). Prediction of IL4 inducing peptides. Clin. Dev. Immunol..

[37] Singh O., Hsu W.L., Su E.C.Y. (2021). ILeukin10Pred: A Computational Approach for Predicting IL-10-Inducing Immunosuppressive Peptides Using Combinations of Amino Acid Global Features. Biology.

[38] Sun P., Ju H., Liu Z., Ning Q., Zhang J., Zhao X., Huang Y., Ma Z., Li Y. (2013). Bioinformatics resources and tools for conformational B-cell epitope prediction. Comput. Math. Methods Med..

[39] Martinelli D.D. (2022). In silico vaccine design: A tutorial in immunoinformatics. Healthc. Anal..

[40] Garg V.K., Avashthi H., Tiwari A., Jain P.A., Ramkete P.W.R., Kayastha A.M., Singh V.K. (2016). MFPPI–Multi FASTA ProtParam Interface. Bioinformation.

[41] Magrane M., Consortium U.P. (2011). UniProt Knowledgebase: A hub of integrated protein data. Database.

[42] McGuffin L.J., Bryson K., Jones D.T. (2000). The PSIPRED protein structure prediction server. Bioinformatics.

[43] Geourjon C., Deléage G. (1995). SOPMA: Significant improvements in protein secondary structure prediction by consensus prediction from multiple alignments. Bioinformatics.

[44] Yang Z., Zeng X., Zhao Y., Chen R. (2023). AlphaFold2 and its applications in the fields of biology and medicine. Signal Transduct. Target. Ther..

[45] Adiyaman R., Edmunds N.S., Genc A.G., Alharbi S.M.A., McGuffin L.J. (2023). Improvement of protein tertiary and quaternary structure predictions using the ReFOLD refinement method and the AlphaFold2 recycling process. Bioinform. Adv..

[46] Jumper J., Evansm R., Pritzel A., Green T., Figurnov M., Ronneberger O., Tunyasuvunakool K., Bates R., Žídek A., Potapenko A. (2021). Highly accurate protein structure prediction with AlphaFold. Nature.

[47] Heo L., Park H., Seok C. (2013). GalaxyRefine: Protein structure refinement driven by side-chain repacking. Nucleic Acids Res..

[48] Lee G.R., Won J., Heo L., Seok C. (2019). GalaxyRefine2: Simultaneous refinement of inaccurate local regions and overall protein structure. Nucleic Acids Res..

[49] Laskowski R.A., MacArthur M.W., Thornton J.M. (1998). Validation of protein models derived from experiment. Curr. Opin. Struct. Biol..

[50] Colovos C., Yeates T.O. (1993). Verification of protein structures: Patterns of nonbonded atomic interactions. Protein Sci..

[51] Wiederstein M., Sippl M.J. (2007). ProSA-web: Interactive web service for the recognition of errors in three-dimensional structures of proteins. Nucleic Acids Res..

[52] Yakubu A., De Donato M., Imumorin I.G. (2017). Modelling functional and structural impact of non-synonymous single nucleotide polymorphisms of the DQA1 gene of three Nigerian goat breeds. S. Afr. J. Anim. Sci..

[53] Kwofie S.K., Dankwa B., Odame E.A., Agamah F.E., Doe L.P.A., Teye J., Agyapong O., Miller W.A., Mosi L., Wilson M.D. (2018). In Silico Screening of Isocitrate Lyase for Novel Anti-Buruli Ulcer Natural Products Originating from Africa. Molecules.

[54] Kumar M., Rathore R.S. (2025). RamPlot: A webserver to draw 2D, 3D and assorted Ramachandran (φ, ψ) maps. J. Appl. Crystallogr..

[55] Oostenbrink C., Villa A., Mark A.E., Van Gunsteren W.F. (2004). A biomolecular force field based on the free enthalpy of hydration and solvation: The GROMOS force-field parameter sets 53A5 and 53A6. J. Comput. Chem..

[56] Rapin N., Lund O., Castiglione F. (2011). Immune system simulation online. Bioinformatics.

[57] Pettersen E.F., Goddard T.D., Huang C.C., Meng E.C., Couch G.S., Croll T.I., Morris J.H., Ferrin T.E. (2021). UCSF ChimeraX: Structure visualization for researchers, educators, and developers. Protein Sci..

[58] Yan Y., Zhang D., Zhou P., Li B., Huang S.Y. (2017). HDOCK: A web server for protein–protein and protein–DNA/RNA docking based on a hybrid strategy. Nucleic Acids Res..

[59] Yan Y., Tao H., He J., Huang S.Y. (2020). The HDOCK server for integrated protein–protein docking. Nat. Protoc..

[60] De Beer T.A.P., Berka K., Thornton J.M., Laskowski R.A. (2014). PDBsum additions. Nucleic Acids Res..

[61] Akhtar H., Akhtar S., Jan S.U., Khan A., Us Sahar N., Zaidi S.S., Qadri I. (2013). Over expression of a synthetic gene encoding interferon lambda using relative synonymous Codon usage bias in Escherichia coli. Pak. J. Pharm. Sci..

[62] Grote A., Hiller K., Scheer M., Münch R., Nörtemann B., Hempel D.C., Jahn D. (2005). JCat: A novel tool to adapt codon usage of a target gene to its potential expression host. Nucleic Acids Res..

[63] Zhang Q., Wang P., Kim Y., Haste-Andersen P., Beaver J., Bourne P.E., Bui H.H., Buus S., Frankild S., Greenbaum J. (2008). Immune epitope database analysis resource (IEDB-AR). Nucleic Acids Res..

